# Successful Integration of EN/ISO 13606–Standardized Extracts From a Patient Mobile App Into an Electronic Health Record: Description of a Methodology

**DOI:** 10.2196/40344

**Published:** 2022-10-12

**Authors:** Santiago Frid, Maria Angeles Fuentes Expósito, Inmaculada Grau-Corral, Clara Amat-Fernandez, Montserrat Muñoz Mateu, Xavier Pastor Duran, Raimundo Lozano-Rubí

**Affiliations:** 1 Medical Informatics Unit Hospital Clínic de Barcelona Barcelona Spain; 2 Universitat de Barcelona Barcelona Spain; 3 FundiSYS Barcelona Spain; 4 mHealth Observatory Hospital Clínic de Barcelona Barcelona Spain; 5 Fundació Clínic per a la Recerca Biomèdica Barcelona Spain; 6 Oncology Unit Hospital Clínic de Barcelona Barcelona Spain

**Keywords:** health information interoperability, mobile app, health information standards, artificial intelligence, electronic health records, machine learning

## Abstract

**Background:**

There is an increasing need to integrate patient-generated health data (PGHD) into health information systems (HISs). The use of health information standards based on the dual model allows the achievement of semantic interoperability among systems. Although there is evidence in the use of the Substitutable Medical Applications and Reusable Technologies on Fast Healthcare Interoperability Resources (SMART on FHIR) framework for standardized communication between mobile apps and electronic health records (EHRs), the use of European Norm/International Organization for Standardization (EN/ISO) 13606 has not been explored yet, despite some advantages over FHIR in terms of modeling and formalization of clinical knowledge, as well as flexibility in the creation of new concepts.

**Objective:**

This study aims to design and implement a methodology based on the dual-model paradigm to communicate clinical information between a patient mobile app (Xemio Research) and an institutional ontology-based clinical repository (OntoCR) without loss of meaning.

**Methods:**

This paper is framed within Artificial intelligence Supporting CAncer Patients across Europe (ASCAPE), a project that aims to use artificial intelligence (AI)/machine learning (ML) mechanisms to support cancer patients’ health status and quality of life (QoL). First, the variables “side effect” and “daily steps” were defined and represented with EN/ISO 13606 archetypes. Next, ontologies that model archetyped concepts and map them to the standard were created and uploaded to OntoCR, where they were ready to receive instantiated patient data. Xemio Research used a conversion module in the ASCAPE Local Edge to transform data entered into the app to create EN/ISO 13606 extracts, which were sent to an Application Programming Interface (API) in OntoCR that maps each element in the normalized XML files to its corresponding location in the ontology. This way, instantiated data of patients are stored in the clinical repository.

**Results:**

Between December 22, 2020, and April 4, 2022, 1100 extracts of 47 patients were successfully communicated (234/1100, 21.3%, extracts of side effects and 866/1100, 78.7%, extracts of daily activity). Furthermore, the creation of EN/ISO 13606–standardized archetypes allows the reuse of clinical information regarding daily activity and side effects, while with the creation of ontologies, we extended the knowledge representation of our clinical repository.

**Conclusions:**

Health information interoperability is one of the requirements for continuity of health care. The dual model allows the separation of knowledge and information in HISs. EN/ISO 13606 was chosen for this project because of the operational mechanisms it offers for data exchange, as well as its flexibility for modeling knowledge and creating new concepts. To the best of our knowledge, this is the first experience reported in the literature of effective communication of EN/ISO 13606 EHR extracts between a patient mobile app and an institutional clinical repository using a scalable standard-agnostic methodology that can be applied to other projects, data sources, and institutions.

## Introduction

### Importance of Patient-Generated Health Data

Traditionally, physicians were the only actor who registered patient data in health information systems (HISs). In recent years, the focus has shifted toward more active participation by patients in their own health care, particularly by means of patient-generated health data (PGHD) [1].

One relevant source of PGHD are wearables, electronic devices that connect to the body surface of patients and can transmit data regarding many biological variables. The number of such devices that generate valuable data is growing considerably.

Furthermore, patient experience has been progressively incorporated into health care processes with the objective to optimize them. One of the most relevant measures of outcomes is the patient-reported outcome measures (PROMs), which record patients’ perception of disease, including relevant symptoms and emotional distress [2]. In the context of the increasingly adopted value-based health care model, Michael Porter developed a formula: value = (results that matter to the patient)/costs [3,4]. In this model, it is key that patients report the results that matter most to them using indicators provided by PROMs [5].

Increasingly, all of these data come from patient mobile apps, and they need to be integrated into HISs for their use in the caregiving process (primary use) or for research purposes (secondary use). However, given the large number of HISs that coexist even within a single health organization, this proves to be highly challenging.

### Interoperability in Health Information Systems

To share clinical information in such a way that it can be unequivocally interpreted, both syntactically and semantically, by 2 or more systems, a common health information standard must be used.

European Norm/International Organization for Standardization (EN/ISO) 13606 is a health information standard that seeks to define a rigorous and stable architecture for communicating health records of a single patient, preserving the original clinical meaning. It is based on a dual model proposed by OpenEHR [6] that includes a reference model (with the necessary components, and their constraints, to represent electronic health record [EHR] extracts) and an archetype model (for the formalization of the clinical domain concepts according to the reference model) [7,8]. Thus, EN/ISO 13606 was designed for the exchange of EHR extracts with full meaning and a high compatibility with OpenEHR [9].

The Fast Healthcare Interoperability Resources (FHIR) standard was developed by Health Level 7 (HL7) with the intention to use modern communication standards for the agile creation of health data communication infrastructures [10]. FHIR’s 80/20 rule (focus on 20% of the requirements that satisfy 80% of the interoperability needs) centers on simplicity rather than completeness. FHIR also provides a health information standard to Substitutable Medical Applications and Reusable Technologies (SMART), a framework that enables medical apps to be written once and run unmodified across different health care information technology (IT) systems [11].

EN/ISO 13606’s advantages over FHIR in terms of modeling and formalization of clinical knowledge, as well as flexibility in the creation of new concepts, suggest it could play a role in the communication of EHR extracts with mobile apps, despite the limited existing evidence. This could be particularly useful in complex scenarios of health data exchange between nodes [12].

### The ASCAPE Project

This paper is framed within the Artificial intelligence Supporting CAncer Patients across Europe (ASCAPE) project, where breast and prostate cancer, 2 of the most prevalent types of cancer, are considered [13]. One of the main purposes of the project is to use powerful artificial intelligence (AI)/machine learning (ML) mechanisms to support cancer patients’ health status and quality of life (QoL) in 4 different pilots [14,15].

Within the ASCAPE project, clinical partners identified previously validated questionnaires used to capture different QoL issues for both types of cancer. AI-based models ingest data from such questionnaires, as well as data regarding daily activity, side effects, and physicians’ interventions, to predict and suggest improvements in patient QoL issues. Hence, ASCAPE prospectively investigates an AI-based approach toward a personalized follow-up strategy for cancer patients focusing on their QoL issues.

The approach chosen in the project to properly process sensitive medical data is federated learning (FL), a decentralized ML technique where local data are used to train shared global models with a central server, keeping the sensitive data locally.

### Objectives

The aim of this study was to design and implement a methodology based on the dual model paradigm in order to communicate clinical information between a patient mobile app and an institutional clinical repository, without loss of meaning. This implies a series of specific objectives:

- To conceptually represent information regarding daily activity and side effects by means of ontologies
- To define a set of archetypes based on EN/ISO 13606 for the standardization and consolidation of patient data in clinical repositories
- To create a scalable conversion module for mobile apps, within the Hospital Clínic de Barcelona’s (HCB) environment, to transform local data and generate EN/ISO 13606–compliant EHR extracts
- To validate the methodology through the successful generation and integration of EHR extracts sent from Xemio Research, a patient mobile app, into the institutional ontology-based clinical repository, OntoCR.

## Methods

### Ethical Considerations

This study was approved by the Hospital Clínic de Barcelona Ethics Committee for Investigation with Drugs (HCB/2020/0971).

### Systems and Servers

#### OntoCR

Traditionally, HISs were developed with a focus on financial and administrative activities, whereas clinical data have been merely translated from paper records to electronic databases. Clinical concepts and the relationships between them have been poorly developed.

OntoCR is an ontology-driven clinical repository conforming to the EN/ISO 13606 standard that uses ontologies for different purposes [16,17]. On the one hand, they define a conceptual architecture centered on the representation of the clinical process and clinical knowledge. By representing a metamodel of health information standards, classifications, and terminologies, OntoCR can also achieve syntactic and semantic interoperability between different HISs. On the other hand, OntoCR uses an ontology that defines the available elements that can be used to build an app. These elements are used by portlets to create a graphical user interface (GUI) deployed in Liferay [18], thus allowing users to access, visualize, enter, and modify structured data through a web-based clinical workstation. OntoCR is linked to the HCB’s EHRs (SAP) using the patient ID, and it can be accessed via SAP or its own website.

#### Xemio Research

Xemio Research was developed for breast cancer patients, providing them with proper information, allowing the tracking of symptoms, and collecting physical activity data from its users on a daily basis (steps, time of activity, and calories). The deployment of Xemio Research’s backend takes place within the gated area of the HCB, with a dedicated server (CentOS Linux) whose database is modeled object-oriented in PostgresDB without normalized codes for secondary effect or activity references, just literals names in Spanish.

Xemio Research is published in Apple App Store and Google Play Store, with access restricted to study participants. The app was installed on the patient’s phone by the field researcher during the first visit, where the patient provided signed informed consent. This generated a Xemio Research ID, which was then registered and linked to the ASCAPE ID in OntoCR by the field researcher.

#### ASCAPE Local Edge

**Figure 1:**
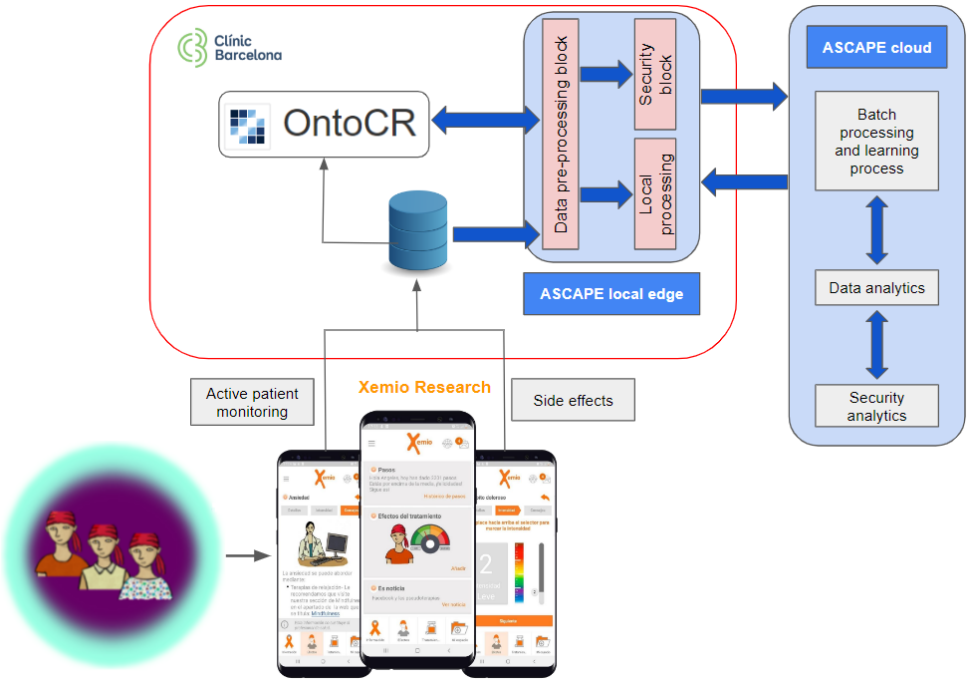
Information systems within the ASCAPE project. Patients register side effects in Xemio Research, which also tracks patients’ daily steps. These data are standardized using a conversion module within the HCB environment (see the Methodology section, step 3), and it is both stored in the OntoCR and sent to ASCAPE Local Edge, which generates and updates ASCAPE’s AI predictive models, which are shared and evaluated in its accuracy in the federated node. AI: artificial intelligence; ASCAPE: Artificial intelligence Supporting CAncer Patients across Europe; HCB: Hospital Clínic de Barcelona.

Due to the sensitive nature of real patient data and the security and data treatment requirements of the project, the ASCAPE architecture was implemented in a dedicated server (ASCAPE Local Edge) within the HCB’s environment, supervised by the local IT department (Figure 1).

This architecture was deployed using Kubernetes (k8s) [19], an open source software that accelerates the implementation and administration of containers on a large scale. These containers maintain the microservices needed for the functioning of the project; the processes of data extraction, transformation, and load (ETL); the normalization of retrospective data provided by the HCB; patient anonymization; and predictions offered by AI models. The aforementioned normalization of local data is performed by identifying variables of interest and transforming them to the ASCAPE Common Data Model and HL7 FHIR [15], thus generating a uniform ASCAPE-standardized database for training data sets to feed the AI engines. Furthermore, Local Edge generates and updates ASCAPE’s AI predictive models [14], which are shared and evaluated in its accuracy in the federated node.

### Methodology

The methodology comprises a series of steps to achieve successful sharing of standardized clinical information between a patient mobile app and an institutional clinical repository.

#### Step 1: Definition of Variables to Communicate and Creation of EN/ISO 13606 Archetypes

The first step in the methodology is to define clinical variables that need to be communicated through EHR extracts. Since this study was framed within the ASCAPE project, we identified variables that needed to be registered and could be recorded with Xemio Research:

- Daily activity: date, steps, calories, and duration
- Side effects: date, finding, value, and severity

To share information standardized with EN/ISO 13606, archetypes that define the chosen variables must be created. EN/ISO 13606’s reference model has multiple components, including the *entry* (“a result of one clinical action, one observation, one clinical interpretation, or one intention”) and its *elements* (“The leaf node of the EHR hierarchy, containing a single data value”).

**Figure 2:**
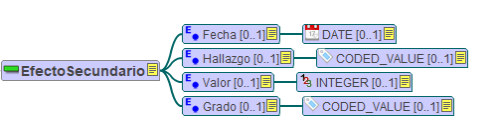
Mindmap of the “side effect” archetype (in Spanish), edited with LinkEHR. The “side effect” entry has 4 elements: date, finding, value, and severity.

Figure 2 shows a mindmap created with the LinkEHR tool [20] of the “side effect” entry archetype. Data types used are those established by the reference model.

#### Step 2: Creation of Ontologies

Once the archetypes are generated, the clinical concepts defined by them must be represented in both systems (mobile app and clinical repository). The functionalities needed to record these variables had already been developed in Xemio Research. For OntoCR, the Medical Informatics Unit at the HCB created the corresponding ontologies to represent these concepts.

**Figure 3:**
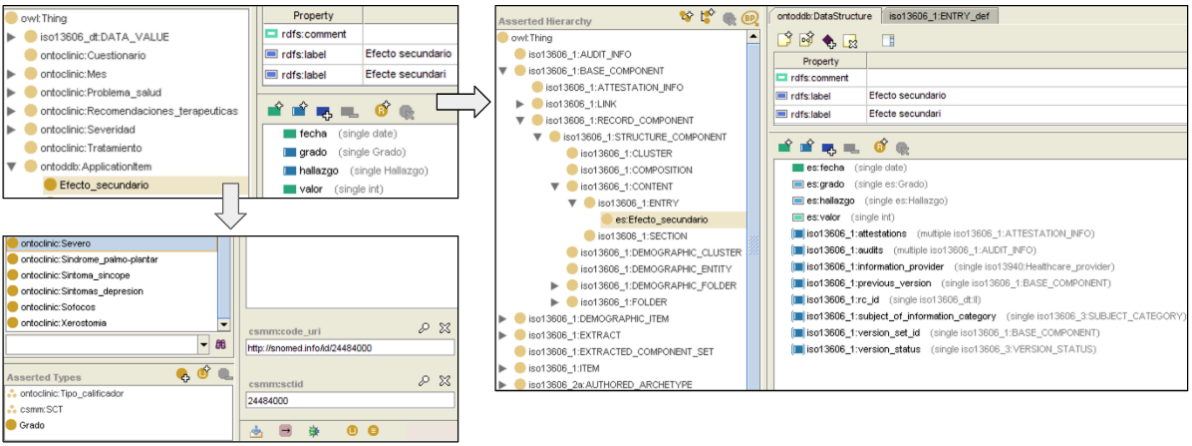
Ontologies of “side effect” modeled locally (upper left) and with EN/ISO 13606 (right) and modeling of the concept “severe” using the international edition of SNOMED CT (lower left), all of them in Spanish and edited with Protégé. EN/ISO: European Norm/International Organization for Standardization; SNOMED CT: Systematized Nomenclature of Medicine – Clinical Terms.

A locally developed ontology named Ontoclinic already had a representation of most of the clinical findings that would be used for this project. Hence, the remaining concepts were modeled and added to Ontoclinic, which was later imported into the ASCAPE ontologies. Ontoclinic also includes metaclasses that represent standard classifications and terminologies. Thus, by indicating that a given class is an instance of the Systematized Nomenclature of Medicine – Clinical Terms (SNOMED CT) metaclass, it allows the normalization of concepts (see Figure 3). Both *finding* and *severity* were coded with the international edition of SNOMED CT using this approach.

Afterward, both Xemio Research and OntoCR had to model local concepts following the standard. In the first case, this was performed by a conversion module in Local Edge, independent from the app. This component is configured by a text document in JSON format that contains the SNOMED CT codes for each side effect and its severity. The procedure was developed in Python, and it transforms, conceptualizes, and generates daily EN/ISO 13606 EHR extracts with the data of Xemio Research users.

In OntoCR, the modeling was performed by means of ontologies. The HCB Medical Informatics Unit created an ontology that incorporates both EN/ISO 13606 reference and archetype models, enabling the capability of representing clinical data that conform to the standard. Therefore, new ontologies of each entry were created, where the concepts defined in the archetypes were mapped to the EN/ISO 13606 structure.

**Figure 4:**
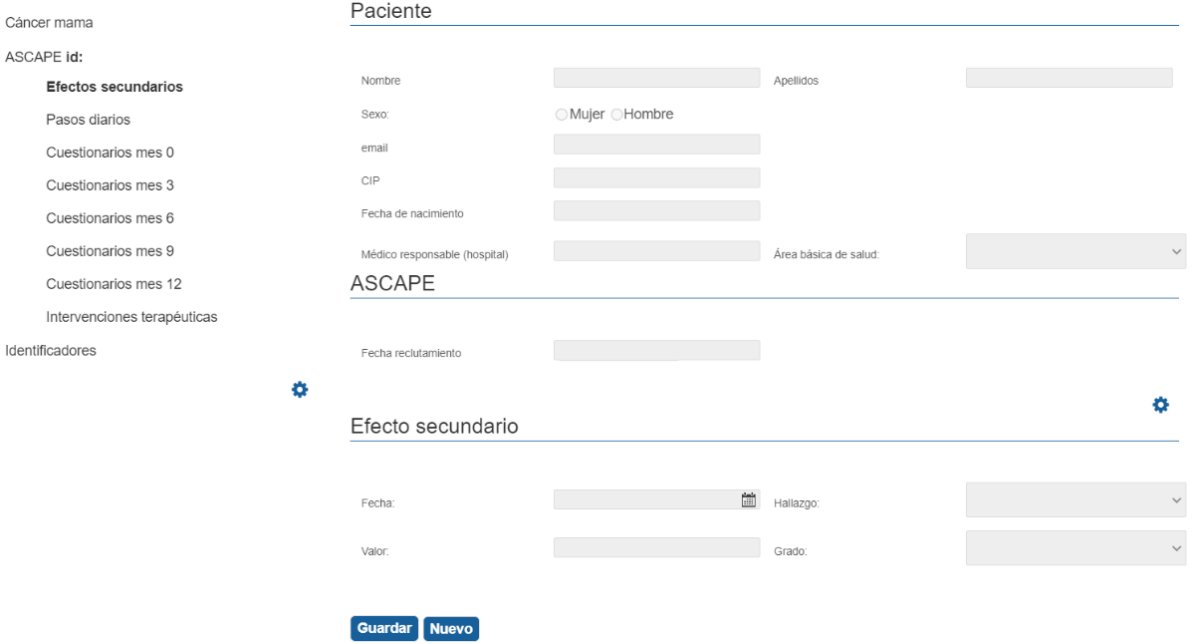
OntoCR GUI for physicians. The ontology modeling the clinical variables is visualized as a web-based structured form. The side effects menu item is selected in the hierarchical menu on the left side of the image. The right side of the image shows the properties regarding patient information, ASCAPE recruitment date, and side effects. ASCAPE: Artificial intelligence Supporting CAncer Patients across Europe; GUI: graphical user interface.

Figure 3 shows the ontological modeling of concepts described in steps 1 and 2. The upper-left image displays the Secondary_effect class of the Ontoclinic ontology, with its properties *date*, *severity*, *finding*, and *value*. The lower-left image shows the modeling of the Ontoclinic Severe class with SNOMED CT, which was performed by making the concept an instance of the SCT metaclass, thus allowing its binding to a code Uniform Resource Identifier (URI) and a concept ID. Finally, the right image displays the Secondary_effect class modeled with EN/ISO 13606 as a subclass of EN/ISO 13606 ENTRY, therefore inheriting properties of its superclass. Once the ontologies that represent the clinical concepts are created, they are uploaded to OntoCR (Figure 4), where they will be ready to receive instantiated patient data.

#### Step 3: Communication of Standardized Extracts

After the variables were defined, represented, and standardized in both systems, extracts were ready to be communicated. Xemio Research has integrated services that transmit extracts with pseudo-anonymized data of either side effects or daily activity collected by the app to an Application Programming Interface (API) in OntoCR, which allows the insertion of extracts into the ontology. This way, instantiated data of patients are stored in OntoCR.

Regarding data security and privacy, Xemio Research generated extracts with anonymous identifiers that were assigned to the patients during recruitment. OntoCR stores the information of both Xemio Research IDs and ASCAPE IDs, so it can integrate the data from the extracts with the rest of the clinical records. Therefore, there is no need for the app to receive data from the hospital’s HIS, which is why communication between Xemio Research and OntoCR is unidirectional. This ensures the confidentiality of the real patient data that are managed.

**Figure 5:**
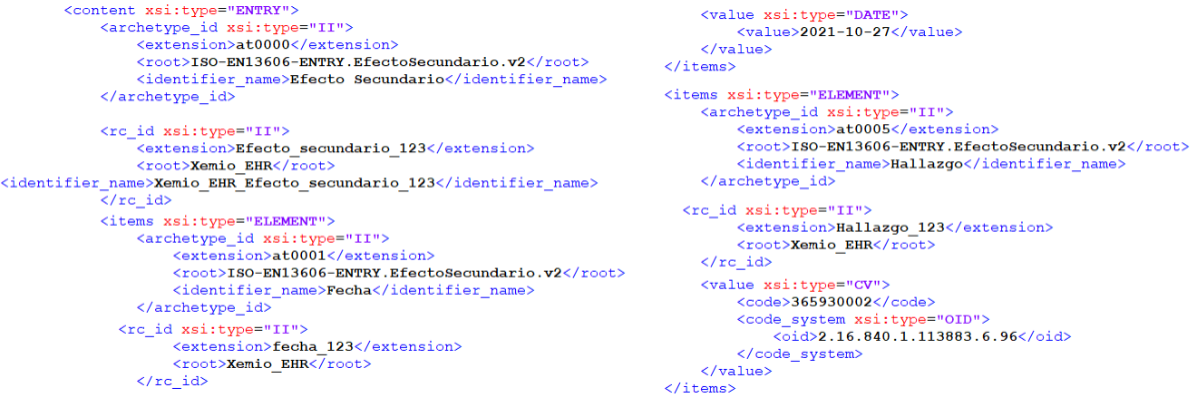
Example of a deidentified EHR extract of side effects. EHR: electronic health record.

An example of an EN/ISO 13606 EHR extract of side effects is displayed in Figure 5, where the “Wakefulness” finding (coded with the SNOMED CT concept ID 365930002) is recorded.

**Figure 6:**
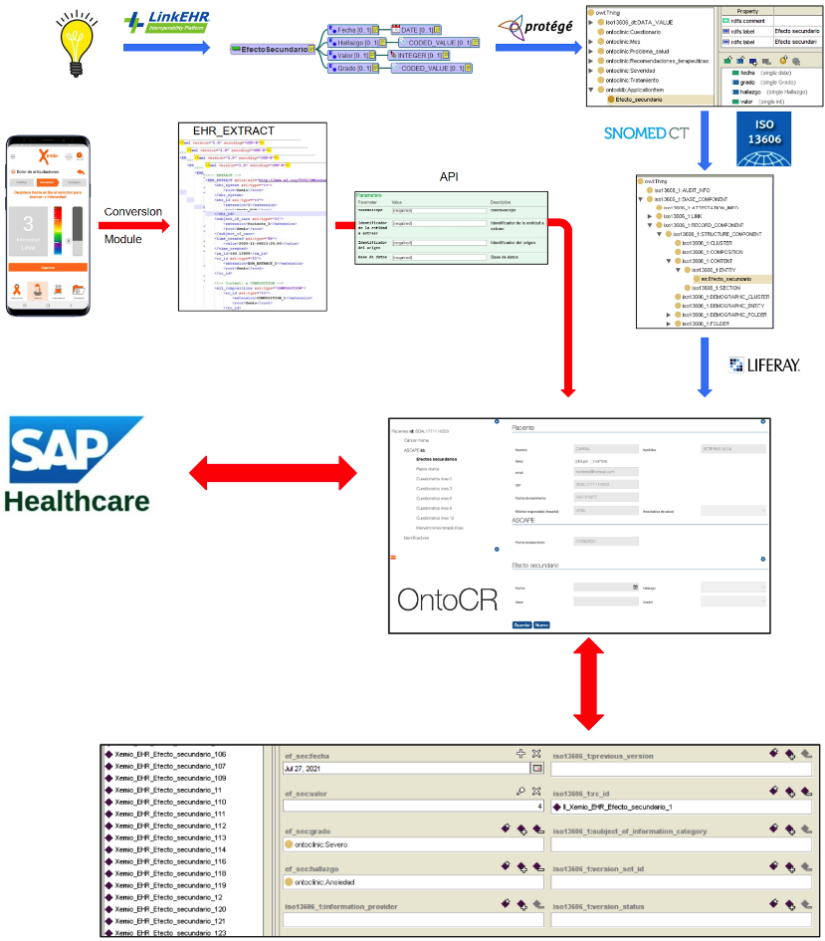
Overview of the process of knowledge modeling and extract communication and integration into OntoCR. Blue arrows indicate knowledge-related processes, while red arrows indicate data-related processes. API: Application Programming Interface; EHR: electronic health record; ISO: International Organization for Standardization; SNOMED CT: Systematized Nomenclature of Medicine – Clinical Terms.

Figure 6 shows an overview of the process of knowledge modeling and extract communication between Xemio Research and OntoCR. Archetypes created with LinkEHR based on clinical concepts are used as templates to model knowledge in ontologies using Protégé. The addition of ontological layers that contain the metamodels of terminologies, such as SNOMED CT, and health information standards, such as EN/ISO 13606, allow for semantic interoperability of the information. These ontologies, without instantiated data yet, are uploaded to OntoCR.

Patients enter information on Xemio Research, which normalizes it through a conversion module, thus creating EN/ISO 13606 EHR extracts. These extracts are sent to the API of OntoCR, which inserts patient data into the ontology. The lower image displays a list of instances of side effects, with the corresponding values of the properties *date*, *value*, *severity*, and *finding* entered in Xemio Research by the patient. Furthermore, an instance of the rc_id EN/ISO 13606 property was inserted, indicating the unique identifier by which this instance is referenced in the EHR system.

The process for developing the communication of extracts started in November 2020 and finished in March 2022, with effective deployment in a production environment. On March 14, 2022, all EHR extracts corresponding to retrospective data were sent, and thereafter, extracts were sent daily.

## Results

### EN/ISO 13606 EHR Extracts

We achieved effective communication of EN/ISO 13606–standardized EHR extracts between a mobile app for patients, Xemio Research, and an institutional clinical repository, OntoCR.

In our study pilot, 62 patients were allocated to use Xemio Research. There were 12 (19.4%) dropouts: 7 (58%) due to a lack of response to questionnaires, 2 (17%) due to medical issues, 2 (17%) lost to follow-up, and 1 (8%) for personal reasons. Furthermore, 3 (4.8%) patients never used the app, leading to a total of 47 (75.8%) users.

Table 1 shows the number of each type of extracts exchanged between December 22, 2020, and April 4, 2022, and the number of patients they pertain to.

When comparing the extracts to the data registered in both Xemio Research and OntoCR databases, no missing or unclear data were detected in the process for the study cohort.

**Table 1:** Number of extracts communicated throughout the study.

EHR^a^ archetype	Extracts (N=1100), n (%)	Patients (N=47), n (%)
Side effects	234 (21.3)	34 (72.3)
Daily activity	866 (78.7)	38 (80.9)

^a^EHR: electronic health record.

### Archetypes and Ontologies

Furthermore, the methodology created for this project resulted in a series of deliverables within each step of the process. First, the creation of EN/ISO 13606–standardized archetypes allows the reuse of clinical information for the variables considered in this study: daily activity (date, steps, calories, and duration) and side effects (date, finding, value, and severity).

In addition, by creating ontologies that represent the aforementioned clinical variables and integrating them into OntoCR, we continue to extend the knowledge representation of our ontology-based clinical repository.

## Discussion

### Principal Findings

We describe a methodology for communicating EN/ISO 13606 EHR extracts between a patient mobile app and an ontology-based clinical repository. Standardized information regarding side effects or daily activity of patients enrolled into Xemio Research in the study was effectively communicated.

EN/ISO 13606 was chosen for this project because of the operational mechanisms it offers for data exchange and its advantages regarding modeling of clinical knowledge and flexibility in the creation of new concepts, which is also why it was used in the first place to extend OntoCR’s metamodel with the incorporation of the reference and archetype models of the standard. However, due to the flexibility and standard-agnostic nature of our methodology, there is complete independence regarding any specific standard. Thus, we are able to carry out transformations between health information standards with minimum use of resources and without the need for changes in the database structure.

LinkEHR offers the possibility to create clinical information models using multiple health information standards (EN/ISO 13606, OpenEHR, FHIR) as well as terminologies and classifications (SNOMED CT, International Classification of Diseases 10th Revision [ICD-10], Logical Observation Identifiers Names and Codes [LOINC]), all of which can also be incorporated into OntoCR by creating corresponding metamodel ontologies. The API that inserts instantiated patient data into the repository is prepared to receive any EN/ISO 13606 EHR extract, and it can be extended to incorporate other standards as well. All this facilitates the application of our methodology to other projects and institutions.

### Single vs Dual Models and Semantic Interoperability in Health Care

Health information interoperability is one of the requirements for the continuity of health care [21]. The dual model allows the separation of knowledge and information in EHR systems, with the consequent possibility of extending the concept model without the need for specific developments and introducing new concepts when the system is already implemented [22]. With the use of formal information models built from common components and linked to standard terminologies [23], 2 systems can achieve semantic interoperability without prior agreement [24,25].

Single, nonstandardized models require the development of specific interfaces to communicate information with other systems. In a context where there is a growing number of information systems within each health organization, many of which come from mobile devices of both patients and physicians, the scalability of this approach is considerably reduced. These difficulties are even greater when considering the communication of health information between different organizations.

The benefits of standardizing EHR data are not limited to primary use. The reuse of clinical data for secondary purposes, such as investigation in both single- and multicenter studies, requires formal information models in order to make data unequivocally understandable and reproducible [26].

### Comparison With Prior Work

There are reports in the literature of standard-agnostic approaches similar to ours, which enable a semantically interoperable clinical data landscape. Gaudet-Blavignac et al [27] propose a 3-pillar strategy based on a multidimensional encoding of concepts, a resource description framework (RDF)–based storage and transport of the instances of these concepts, and a conversion of the RDF to any target data model. Likewise, the INFOBANCO project of the Madrid Region in Spain [28] aims to create a platform for the management, persistence, exchange, and reuse of health data, contemplating 2 types of outputs: interoperability (HL7 FHIR, EN/ISO 13606, Clinical Data Interchange Standards Consortium [CDISC] [29]) and persistence (OpenEHR, i2b2, Observational Medical Outcomes Partnership Common Data Model [OMOP CDM]). It uses a standard-agnostic design that seeks to apply each health information standard for the purpose it was intended to, offering multiple interoperability and exploitation services suited for specific use cases [12]. However, these projects focus on the creation of interoperable platforms for different purposes, but they do not include a strategy for integrating information coming from mobile apps.

Other groups have reported the use of the SMART on FHIR framework to integrate PGHD from mobile apps into EHRs [30-33]. This framework enables medical apps to be written once and run unmodified across different health care IT systems and has proven to be an effective approach for interoperability. FHIR offers operational mechanisms for data exchange, but unlike EN/ISO 13606, it lacks the capacity to build new concepts based on specific requirements [12], which limits its flexibility to adapt to new scenarios.

### Strengths and Limitations

There are strengths to this study that are worth mentioning. First, the 3 main software programs used (LinkEHR, Protégé, and Liferay) are open source, which makes our methodology accessible to low-income areas as well as institutions with limited funding for such projects. Moreover, the aforementioned flexibility and standard-agnostic nature of our methodology define a considerable scalability. The knowledge representation can be adjusted to different contexts with little resources, just by creating new archetypes, modeling the clinical concepts, and mapping them to the corresponding structure of EN/ISO 13606. If a different health information standard is to be used, its metamodel must be represented with ontologies, and both the conversion module and the API need to be adjusted.

With a few exceptions, such as the experience reported by Zenteno et al [34], there is limited evidence in the literature regarding the effective communication and integration of EN/ISO 13606–standardized extracts from a mobile app into an EHR. In addition, to the best of our knowledge, ours is the first experience that does so with data coming from a patient mobile app. Given EN/ISO 13606’s advantages over FHIR in terms of modeling and formalization of clinical knowledge and flexibility in the creation of new concepts, our approach proves to be quite innovative in the communication of EHR extracts with mobile apps.

This study also has some limitations. First, even though there is a log file in the server that registers the extracts that are sent, there is no alarm that notifies us when the process is not working. Therefore, this maintenance and update of the system still depends on manual processes. Furthermore, the ontology-based approach requires trained staff and an initial development that involves the allocation of resources in terms of personnel, funds, and time, which can limit the extensibility of the methodology to other contexts.

### Next Steps

Regarding next steps of the project, we are in the process of integrating a dashboard into OntoCR, which will display the AI-based predicted variation in the QoL issues according to the interventions carried out by physicians. This will help physicians with their clinical decision-making when evaluating treatment alternatives for breast cancer patients.

Furthermore, we are working on extending the integration of extracts to other functionalities in Xemio Research, and later, we plan to do so with other mobile apps used within the HCB ecosystem.

### Conclusion

This study describes a novel methodology for the successful communication of standardized EHR extracts from a patient mobile app with an ontology-based clinical repository linked to an EHR. Its flexibility and standard-agnostic nature provide significant scalability to adapt to different contexts, situations, and information systems, while the use of open source software facilitates its transferability to other institutions. Our approach allows the integration of data coming from different sources into HISs for them to be used in the caregiving process (primary use) or for investigation purposes (secondary use). To the best of our knowledge, this is the first study to achieve effective communication and integration of EN/ISO 13606–standardized extracts from a patient mobile app into an EHR.

## Abbreviations

AI: artificial intelligence
API: Application Programming Interface
ASCAPE: Artificial intelligence Supporting CAncer Patients across Europe
CDISC: Clinical Data Interchange Standards Consortium
EHR: electronic health record
EN/ISO: European Norm/International Organization for Standardization
FHIR: Fast Healthcare Interoperability Resources
FL: federated learning
GUI: graphical user interface
HCB: Hospital Clínic de Barcelona
HIS: health information system
HL7: Health Level 7
ICD-10: International Classification of Diseases 10th Revision
IT: information technology
LOINC: Logical Observation Identifiers Names and Codes
ML: machine learning
OMOP CDM: Observational Medical Outcomes Partnership Common Data Model
PGHD: patient-generated health data
PROM: patient-reported outcome measure
QoL: quality of life
SMART: Substitutable Medical Applications and Reusable Technologies
SNOMED CT: Systematized Nomenclature of Medicine – Clinical Terms
URI: uniform resource identifier
